# Baccharis articulata aqueous extract exerts in vitro antifibrotic effect in hepatic stellate cells by attenuating collagen deposition and TGF-ß1 protein expression

**DOI:** 10.17179/excli2025-8394

**Published:** 2025-05-13

**Authors:** Daiana Daniele Boeff, Markus Berger, Mariana Koetz, Pamela Zanon, Alícia da Costa Pereira, Katyuce de Souza Farias, Carlos Alexandre Carollo, Paula Barros Terraciano, Eduardo Luis Konrath

**Affiliations:** 1 Laboratory of Pharmacognosy, Faculty of Pharmacy, Federal University of Rio Grande do Sul (UFRGS), Porto Alegre, RS, Brazil; 2 Post-Graduation Program in Pharmaceutical Sciences, Federal University of Rio Grande do Sul (UFRGS), Porto Alegre, RS, Brazil; 3 Reproduction and Cellular Pharmacology Lab, Experimental Research Center, General Clinical Hospital (HCPA). Porto Alegre, RS, Brazil; 4 Laboratory of Natural Products and Mass Spectrometry, Federal University of Mato Grosso do Sul (UFMS), Campo Grande, MS, Brazil

**Keywords:** Baccharis articulata, liver fibrosis, chlorogenic acid, extracellular matrix, collagen, plasminogen

## Abstract

*Baccharis articulata *(Lam) Pers. is an herb native to southern Brazil and is widely used in local traditional medicine for weight loss and for the treatment of digestive and liver diseases. However, only a few studies have been conducted to scientifically validate the folk use of this plant. This study assessed the *in vitro *therapeutic effects of an aqueous extract of *B. articulata *and chlorogenic acid on liver fibrosis in murine hepatic stellate cells (HSC; GRX cell line). The decrease in cell proliferation and cytotoxicity, as well as phenotypic reversion by the presence of lipid droplets and reduction in collagen content after seven days of treatment, were evaluated. The mechanisms responsible for the antifibrotic effects of the extract, including the plasminogen activation system, were assessed. from high-performance liquid chromatography coupled with diode array detector and tandem mass spectrometry (HPLC-DAD-MS/MS) data. Twenty-six metabolites were identified in the extract, including flavonoids, phenylpropanoid derivatives, and diterpenes. Treatment with the extract significantly induced the accumulation of lipids in the cytoplasm of cells, indicating that it could revert the HSC phenotype to a quiescent state with no cytotoxic or antiproliferative effects. These findings may be related to the inhibition of the TGF-β1 pathway, a biomarker of liver fibrosis, upregulation of the plasminogen system, and dose-dependent inhibition of plasmin activity. The presence of caffeoylquinic acids seems to be partially related to the extract effect, as chlorogenic acid displayed antiproliferative activity and reduced collagen content in hepatic stellate cells. Considering the unmet need for antifibrotic therapies, the use of medicinal plants to inhibit the proliferation of activated HSC is promising, and this study indicated that the aqueous extract of *B. articulata* has potential therapeutic activity against hepatic fibrosis (see also Figure 1(Fig. 1) graphical abstract).

## Introduction

Liver fibrosis is characterized by excessive and continuous deposition of extracellular matrix (ECM) proteins, particularly collagen types I and III (Borojevic et al., 1985). Infections, alcohol abuse, toxins, and metabolic disorders are among the factors that can induce ECM accumulation in humans, thus altering the balance between the synthesis and degradation of collagen fibers and leading to scar deposition (George et al., 2019[29]). Due to the chronicity of the injury in liver diseases, hepatic fibrosis can result in cirrhosis, a complex disease associated with high rates of mortality and characterized by alterations in the liver parenchyma and vascular architecture (Bataller and Brenner, 2005[7]).

Hepatic stellate cells (HSC) are non-parenchymal liver cells that play key roles in the pathophysiology of liver fibrosis. Under physiological conditions, they exist in a quiescent state and present phenotypes and marker characteristics of adipocytes (Friedman et al., 2008[28], 2010[27]). Following injury, HSC are activated and transdifferentiate into profibrogenic myofibroblast-like cells in response to transforming growth factor (TGF-β1) and platelet-derived growth factor (PDGF) secretion (Li et al., 2008[40]; Tsuchida and Friedman, 2017[57]). In this activated state, the cell proliferation rate is increased, secreting a large amount of ECM proteins, mainly collagen fibers, and contributing to tissue repair in liver diseases (Tsuchida and Friedman, 2017[57]). Finally, this process is perpetuated by the production of pro-inflammatory mediators by activated HSC, resulting in excessive ECM deposition, replacement of normal tissue with non-functional scar tissue, and eventually organ failure (Cogliati et al., 2023[21]). 

Currently, no specific drugs have been approved for the treatment of hepatic fibrosis (Tsuchida and Friedman, 2017[57]) and the use of anti-inflammatory drugs, glucocorticoids, and immunosuppressants is associated with poor efficacy and adverse reactions (Shan et al., 2022[49]). One of the most challenging aspects of hepatic fibrosis treatment is the complexity of the cell activation process and the many factors and steps involved in fibrogenesis (Friedman et al., 2010[27]). Therefore, new antifibrotic therapies that target the inhibition of the activation, proliferation, or synthesis of cytokines and growth factors by activated HSC have been remarkably successful (Bogomolova et al., 2024[9]). Medicinal plants hold significant value in understanding the treatment of liver fibrosis because one advantage of plant-based therapies is the presence of different potential bioactive molecules in herbal preparations (Pelkonen et al., 2014[44]). Consequently, they can act simultaneously on multiple potential targets and mechanisms, which is desirable for the treatment of complex chronic diseases, such as liver fibrosis (Chang and Li, 2020[17]; Zhang et al., 2022[62]). 

Historically, bitter plants such as *Baccharis trimera *(Less.) DC., and *Strychnos pseudoquina* A.St.-Hil. are commonly used in Brazilian folk medicine as digestive tonics to stimulate, clean, and protect liver and gallbladder function (Antunes et al., 2022[2]). *Baccharis articulata *(Lam) Pers (Asteraceae)*, *also known as “carqueja-miúda”, is a native Brazilian plant that has one of the highest bitter taste indices among *Baccharis* species (Budel et al., 2004[14]). The “carquejas” share botanical morphological similarities that include their winged stems (cladodes) and branches (Barroso and Bueno, 2002[4]), which reflect in their common traditional indications for cholagogue and choleretic effects (Lopes and Alvarez Filho, 1997[41]; Simões et al., 1998[52]). Aqueous beverages prepared from the aerial parts of* B. articulata* are widely used in folk medicine to treat diabetes and digestive and hepatic diseases, in addition to being a popular herb used for weight loss in southern Brazil (Dickel et al., 2007[26]).

The aerial parts of* B. articulata *have been shown to display antihyperglycemic, insulin-secretagogue, antiviral, and antimutagenic activities (Kappel et al., 2012[33]; Rodríguez et al., 2011[47]; Torres et al., 2011[56]), and their pharmacological effects are often associated with a high content of flavonoids and other polyphenols (de Oliveira et al., 2003[23], 2014[24]; Vieira et al., 2011[58]). For example, chlorogenic acid (5-*O*-caffeoylquinic acid) and other caffeoylquinic acids identified in the genus* Baccharis *have antioxidant and anti-inflammatory properties (Bagdas et al., 2020[3]; Cariddi et al., 2012[16]; Rodríguez et al., 2011[47]). Although this is a promising species, the possible effects of this plant on hepatic disorders remain unclear. In this context, the present study investigated the possible liver antifibrotic effects of an aqueous preparation of *B. articulata*, reflecting its popular use in humans, in activated murine HSCs (GRX) and the possible mechanisms that have evolved during treatment.

## Material and Methods

### Chemicals and reagents

Low-glucose Dulbecco's modified Eagle medium (DMEM), fetal bovine serum (FBS), penicillin/ streptomycin, EDTA, trypsin, 3-(4,5-dimethylthiazol-2-yl)- 2,5-diphenyltetrazolium bromide (MTT), and trypan blue dye were purchased from Life Technologies (Carlsbad, CA, USA). The chromogenic substrate for plasmin (S2251) was obtained from Chromogenix (Milan, Italy). The primary horseradish peroxidase-conjugated and secondary antibodies PAI-1 (plasminogen activator inhibitor 1) and TGF-β1 (transforming growth factor beta 1) were obtained from Santa Cruz Biotechnology, USA. Chlorogenic acid (98%) and pyrogallol (98 %) standards were purchased from Merck (Darmstadt, Germany) and Sigma-Aldrich (St. Louis, MO, USA), respectively. Water was treated using a Milli-Q water purification system (Millipore, Bedford, MA, USA). All other chemicals and organic solvents used were of highest purity available and were commercially available.

### Collection and botanical authentication 

Plant material was collected in April 2022 from the Porto Alegre Botanical Garden (Porto Alegre, Rio Grande do Sul State, Brazil), and a voucher was deposited under number ICN 204261. Legal access to plant material was obtained by registering this study in the National System for the Management of Genetic Heritage and Associated Traditional Knowledge (SisGen, number A2744AB).

### Preparation of the B. articulata aqueous extract (BAE)

The BAE was prepared by decoction according to popular use based on the preparation of the aqueous extract of *B. trimera* present in the Herbal Medicines National Formulary (Brazil, 2021[13]). Briefly, the extract was obtained by boiling the dried and ground wing stems (0.5 g/ 150 mL distilled water) for 5 min. After reaching room temperature (25 ºC), the extract was filtered and lyophilized. The BAE was stored at - 18º C until further analysis.

### Determination of total polyphenols

The total phenolic content of BAE was determined using the classic Folin-Ciocalteu method (Singleton and Rossi, 1965[53]) with some slight modifications. Briefly, 2 mL of the extract, 1.5 mL of Folin-Ciocalteu reagent, 10 mL of distilled water and 11.5 mL of 15% sodium carbonate were mixed and incubated at room temperature in the dark. After 25 min, the absorbance of the mixed solution was measured at 780 nm wavelength. The total polyphenol content was expressed as milligrams of pyrogallol equivalents per gram of extract.

### HPLC-DAD-MS/MS analysis of BAE

The analysis of BAE was conducted using a Shimadzu Prominence UFLC system coupled with a diode array detector (DAD) and a MicrOTOF-Q III mass spectrometer (Bruker Daltonics). Chromatographic separation was performed on a Kinetex® C18 column (2.6 µm, 150 × 2.1 mm, Phenomenex), with a mobile phase consisting of 0.1 % formic acid in water (solvent A) and acetonitrile (solvent B). Gradient elution was as follows: 0-2 minutes at 3 % B, 2-25 minutes from 3 % to 25 % B, and 25-40 minutes from 25 % to 80% B, followed by an 8-minute column wash and reconditioning. The flow rate was set to 0.3 mL/min, with an injection volume of 1 µL (1 mg/mL extract). The oven temperature was maintained at 50° C. The mass spectrometer was operated in both negative and positive ionization modes, using nitrogen as the nebulizer gas (4 bar) and dry gas (9 L/min), with a capillary voltage of 2.5 kV. Identification of major compounds was based on UV spectra and ESI fragmentation patterns compared to previously reported data and authentic standards.

### Determination of total caffeic acid derivatives content by HPLC-DAD

The quantification of total caffeic acid derivatives in the BAE was performed according to the *B. trimera *monograph presented in the Brazilian Pharmacopeia, 6^th^ ed. (Brazil, 2019[12]). Quantitative analysis was performed using high-performance liquid chromatography (HPLC, Waters Alliance model e2695) coupled with a diode array detector (DAD). Chromatographic separation was conducted using a C18 column (Phenomenex, 150 x 4.6 mm x 4 μm) at room temperature using a gradient elution program at a flow rate of 0.6 mL/min and detection was performed at 325 nm. The mobile phases consisted of (A) ultrapure water (Milli-Q) containing acetonitrile and acidified with trifluoroacetic acid at a proportion of 95:5:0.05 (v/v/v) and (B) acetonitrile. The following linear gradient was applied: 0-30 min, 0-43 % B; 30-35 min, 43-100 % B; 35-36 min, 0-100 % A; 36-42 min, 100 % A. Samples were prepared at a concentration of 5.3 mg/mL, filtered using a 0.22 µm nylon syringe filter (Allcrom), and 10 μL was injected into the chromatographic system. Total caffeic acids were quantified using a calibration curve constructed by three injections of a solution prepared with a chlorogenic acid standard (0.0085, 0.017, 0.035, 0.07, 0.14 and 0.28 mg/mL). Data were acquired using Empower® software, and data processing was performed using Excel (2013). 

### GRX cell culture

The murine hepatic stellate GRX cell line was obtained from the Rio de Janeiro Cell Bank (UFRJ, Rio de Janeiro, Brazil) and kindly donated by Prof. Dr. Fátima Guma (UFRGS, Porto Alegre, Brazil). Cells were cultured in low-glucose DMEM medium supplemented with 10 % FBS and 1 % penicillin/streptomycin in a humidified incubator with 5 % CO_2_ at 37ºC. Following confluence, GRX cells were dissociated with 0.1 %/ 0.01 % trypsin/EDTA and then seeded into new culture flasks or multi-well plates for a maximum of 12 passages.

### Cell viability analysis using the MTT assay

The effects of BAE on cell viability were measured by the MTT assay. Briefly, GRX cells (1 x 10^4^ cells/well) were seeded in 96-well plates for 24 h to obtain confluent monolayers and then treated with medium containing BAE at concentrations from 5 to 250 µg/mL. After 24 h, the media was removed, 100 μL of MTT solution (0.5 mg/mL) was added, and the cells were incubated for 3 hours at 37º C. Following incubation, the supernatant was discarded and the formazan crystals were dissolved in DMSO. The absorbance was measured at 585 nm using a spectrophotometer (SpectraMAX 190 microplate reader).

### Cell viability analysis using the trypan blue exclusion method

The effects of BAE on GRX cell growth were assessed by direct counting in a Neubauer chamber using trypan blue staining to differentiate between viable and nonviable cells. Briefly, GRX cells (1.1 x 10^4^ cells) were seeded in T25 cell culture flasks, treated with 50 and 200 µg/mL of BAE or 50 µg/mL of chlorogenic acid for 24 h, and then harvested. The control group received only the culture medium. Cellular viability was assessed by trypan blue dye exclusion after seven days of incubation. The results are presented as percentages of viable and total cells.

### Lipid droplet detection using the Oil Red O staining 

For cell morphology and lipid droplet detection, cells were seeded in T25 culture flasks (1.0 x 10^4 ^cells/flask) for 24 h and then treated with 50 and 200 µg/mL of BAE for 7 days. After the cells were fixed with 10 % formaldehyde, Oil Red O staining solution was added for 15 min as previously described (Ramírez-Zacarías et al., 1992[46]). Intracellular lipid droplets were examined using an inverted light microscope at magnification of 400x. The droplets were counted using Image J software (available at https://imagej.nih.gov/ij/). Lipid accumulation was calculated as the ratio of the number of stained lipid droplets to total number of cells. 

### Measurement of the collagen content using the Sirius red staining

The collagen content in GRX cells was measured in the supernatants using the Sirius Red staining method (Keira et al., 2004[34]) to evaluate the effects of BAE (10, 50, and 200 µg/mL) and chlorogenic acid (50 µg/mL) after incubation for 2, 4, and 7 days. This method is based on the selective reaction between collagen type I and Sirius Red, forming a dye complex that is directly correlated with collagen content. After incubation, the cell supernatant was collected and incubated with dye diluted in 0.5 M acetic acid to form and precipitate the complex. After incubation, the samples were centrifuged and the unbound dye was removed. The pellets were then dissolved in 0.1 % KOH solution, and the absorbance was read at 540 nm. Each sample was normalized to the relative amount of total protein as measured using the Bradford assay (Bradford, 1976[11]). The results were calculated based on the standard curve concentration of collagen type 1, and collagen levels were expressed as a percentage of mg of collagen per mg of protein.

### Western blot analysis

Total protein was extracted from GRX cells in RIPA buffer containing protease and phosphatase inhibitors. Thirty to 50 µg of proteins were subjected to sodium dodecyl sulfate-polyacrylamide gel electrophoresis (SDS-PAGE) under reducing conditions. The proteins were transferred onto nitrocellulose membranes using a Trans-Blot Turbo Transfer System (Bio-Rad, CA, USA). After blocking, the individual primary horseradish peroxidase-conjugated antibodies (Santa Cruz Biotechnology, USA), PAI-1 (1:500 dilution), and TGF-β1 (1:500 dilution) were used to detect specific proteins. The membrane was subsequently washed thrice with PBS-Tween buffer, after which each blot was incubated with the corresponding secondary antibody for 1 h. Immunoreactive proteins were visualized by ECL detection using an iBright CL750 imaging system (Thermo Fisher Scientific, MA, USA). Expression levels were normalized against β-actin and quantified using the ImageJ software. 

### Plasmin generation assays in GRX cells

The effects of BAE on plasmin generation were measured in the presence of human plasminogen using GRX cell monolayers as a source of plasminogen activator activity according to the previously described method (Zanon et al., 2024[61]). Confluent cells were treated with BA (1, 10, 50, 100, and 200 µg/mL) for 24 h in the presence of plasminogen (0.1 mg/mL). The generated plasmin activity was measured directly on cell monolayers using the S2251 synthetic substrate (0.2 mM). The kinetics of *p*-nitroaniline formation was measured at 405 nm, and enzyme activity was expressed as mOD/min/mg of protein.

### Inhibition of plasmin activity

Human plasmin (10 nM) was pre-incubated at 37^o ^C for 15 min with different concentrations of BAE (1, 10, 50, 100, 200, 400, 800 and 1600 µg/mL) in a reaction medium containing 20 mM Tris-HCl, and 150 mM NaCl (pH 7.4). The remaining plasmin activity was measured by adding a 0.2 mM solution of S-2251 substrate (H-D-Val-Leu-Lys-*p*-nitroanilide) in a volume of 100 µL. The amount of *p*-nitroaniline produced was monitored at 405 nm at intervals of 14 s for 30 min, using a spectrophotometer. Progress curves were obtained by plotting the absorbance values versus time. For all calculations, the initial rate of hydrolysis was chosen to determine the steady-state kinetics (V*max* mode) of the BAE-plasmin interactions. IC_50_ values were obtained by plotting the plasmin residual activity versus BAE concentrations. All data points from the kinetic measurements were fitted using the GraphPad Prism software (GraphPad Software, Inc., San Diego, CA, USA). Data represent the mean of three determinations, each performed in triplicate (Zanon et al., 2024[61]).

### Statistical analysis

The data obtained were analyzed by one-way analysis of variance (ANOVA) followed by Bonferroni's test using the GraphPad Prism software (GraphPad Software, Inc., San Diego, CA, USA). Results are presented as mean ± SEM, and *p* values < 0.05 were considered statistically significant. 

## Results and Discussion

### Annotation and quantification of the metabolites from BAE

**Figure 2:**
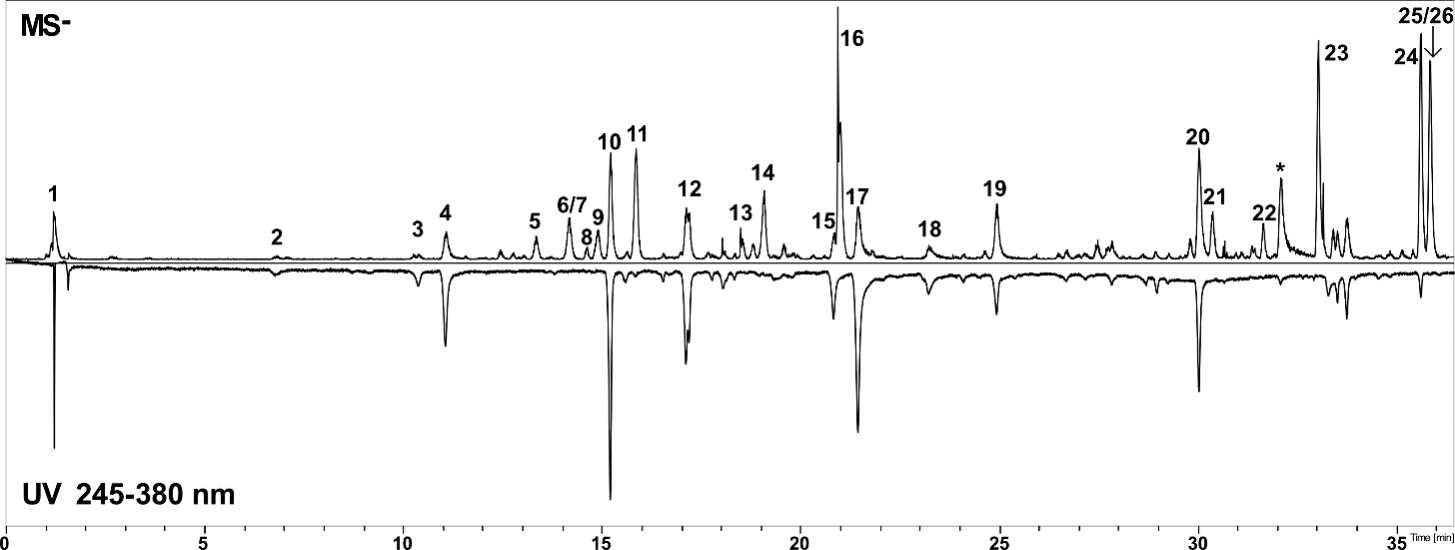
Base peak chromatogram obtained in negative ion mode (MS^-^) and UV chromatogram (245-380 nm) of *Baccharis articulata *aqueous extract (BAE). *: Column contaminants.

Previous studies on *B. articulata *extracts showed the presence of diterpenes, flavonoids, and other phenolic compounds (Dai et al., 1993[22]; de Oliveira et al., 2003[23]). The most representative metabolites belong to the class of caffeoylquinic acids, which are commonly found in several *Baccharis* species (Cariddi et al., 2012[16]). Accordingly, HPLC-DAD-MS/MS analysis of BAE revealed a variety of compounds, which were grouped into three major categories based on their UV spectra, molecular formulas, and MS/MS fragmentation patterns (Fig. 2(Fig. 2), Table 1(Tab. 1)). The most prominent class of compounds detected was phenylpropanoid derivatives, followed by flavonoids, and several putative diterpenes. The phenylpropanoid derivatives were identified and characterized by their typical UV absorption (*λ*_max_ ≈ 291 and 326 nm), corresponding to caffeic acid and its derivatives. Compounds 2, 3, 4, 15, 17, and 18 displayed UV spectra consistent with phenylpropanoid structures, particularly the caffeic acid derivatives. Among them, compounds 3 (caffeic acid, 10.4 min), 4 (5-*O*-*E*-caffeoylquinic acid, 11.0 min), 17 (3,5-dicaffeoylquinic acid, 21.4 min), and 18 (4,5-dicaffeoylquinic acid, 23.2 min) were identified by comparison with the authentic standards, confirming their structures. Caffeic acid and its derivatives exhibit characteristic MS/MS fragment ions, such as m/z 191 and 179, reinforcing their identification as caffeoylquinic acids (Clifford et al., 2003[19], 2005[20]). 

In addition to phenylpropanoid derivatives, flavonoids were also identified in the extract. Compound 10, corresponding to 6,8-di-*C*-hexoside apigenin (15.2 min), and compound 30, corresponding to apigenin (30 min), were detected based on their distinct UV absorption (*λ*_max_ ≈ 270 and 340 nm). The identification of apigenin and its derivatives was further confirmed by their molecular formulas and MS/MS fragmentation patterns, with apigenin displaying fragment ions at m/z 299 and 255. Compound 12 (17.1 min) displayed UV absorption peaks at 271 and 335 nm and produced characteristic MS/MS fragment ions at m/z 503 and 473 consistent with isoschaftoside, a compound that has previously been reported in other *Baccharis* species (Akaike et al., 2003[1]). 

Another important group of compounds identified was putative diterpenes. Compounds 13, 14, 16, 21, 22, 23, 24, 25, and 26 exhibited molecular formulae consistent with those of diterpenes, but no UV absorption was observed for these peaks, suggesting the absence of conjugated double bonds. This lack of UV absorption, combined with their molecular formulae, aligns with previously reported diterpenes in *B. articulata* (Stapel et al., 1980[54]). Despite their consistent fragmentation patterns and molecular formulas, these compounds could not be fully identified because of the absence of reference standards and the limited fragmentation data available in the literature. Therefore, further isolation and structural elucidation are necessary to confirm their identity. In this study, quantification of the total caffeic acid derivatives in BAE was performed using the HPLC-UV-DAD method present in the herbal drug monograph of *B. trimera*, which was included in the Brazilian Pharmacopoeia 6th edition (Brazil, 2019[12]). The relative retention times of caffeoylquinic acids were calculated based on the approximate relative retention time of chlorogenic acid. As a result, all four compounds were detected in BAE, resulting in a total content of 4.3 g%, expressed as chlorogenic acid (0.9 g%), 3,4-dicaffeoylquinic acid (0.8 g%), 3,5-dicaffeoylquinic acid (2.2 g%) and 4,5-dicaffeoylquinic acid (0.4 g%).

Similarly, Rodríguez et al. (2011[47]) reported that the chlorogenic acid content in the aqueous extract of *B. articulata* aerial parts was close to 1.0%, whereas another study conducted by Cariddi et al. (2012[16]) reported an aqueous extract containing 6.2 g% caffeoylquinic acids. Such differences in the contents of caffeic acid derivatives could be explained by the fact that the extracts were obtained differently depending on the place and date of collection of plant material, time, and water temperature used for the extraction method. Additionally, the results of the phytochemical analysis of the total polyphenol content of BAE determined by spectrophotometric assay were expressed as pyrogallol equivalents (PE), and revealed a polyphenol content of 11.4 % (w/w).

### Effects of BAE on the activated phenotype of GRX myofibroblasts

Hepatic fibrosis is a wound-healing process that generally results from chronic and persistent liver injury caused by different etiologic agents, including toxins, hepatitis B and C, and alcohol abuse (Roehlen et al., 2020[48]). This reversible event normally causes an extensive inflammatory response, inevitably leading to scarring and subsequent liver cirrhosis followed by hepatocellular death and liver failure (Lepreux and Desmoulière, 2015[38]). Notably, cirrhosis is a major global health problem, and there are currently no effective therapies to minimize or reverse disease progression (Ginès et al., 2021[30]), justifying the search for new active compounds.

HSC are a primary source of activated myofibroblasts, which play an essential role in triggering fibrinogenesis (Zhang et al., 2021[64]). After activation, these cells lose intracellular lipid droplets and contribute to excessive extracellular matrix deposition, as observed in the pathology of liver fibrosis (Kitano and Bloomston, 2016[36]). Therefore, understanding the biochemical and molecular mechanisms underlying this process is essential for the development of novel therapeutic targets. In this sense, two possible pathways have been described to decrease liver fibrosis, among them the inhibition of cell proliferation and the reversal of cell phenotype for the hepatic stellate cells (Bastos et al., 2023[6]). 

**Figure 3:**
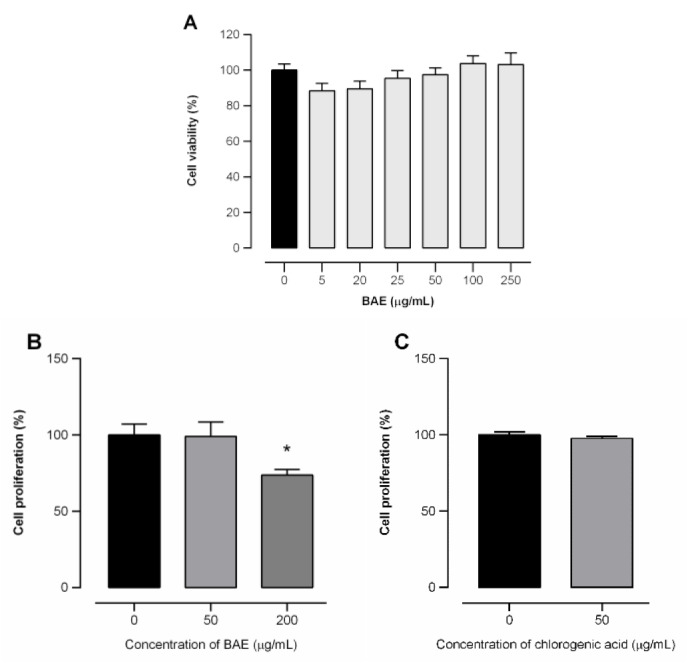
Cell viability of murine hepatic stellate (GRX) cells treated with *Baccharis articulata *aqueous extract (BAE, 0 - 250 µM) for 24 h as measured by the MTT assay (panel A). Effect of BAE (0- 200 µM, panel B) and chlorogenic acid (50 µM, panel C) on GRX cell proliferation after seven days of treatment. Results are expressed as mean ± SEM (n = 4) analyzed by ANOVA followed by Bonferroni's post-hoc test. * *p* < 0.05: control vs. treated cells.

**Figure 4:**
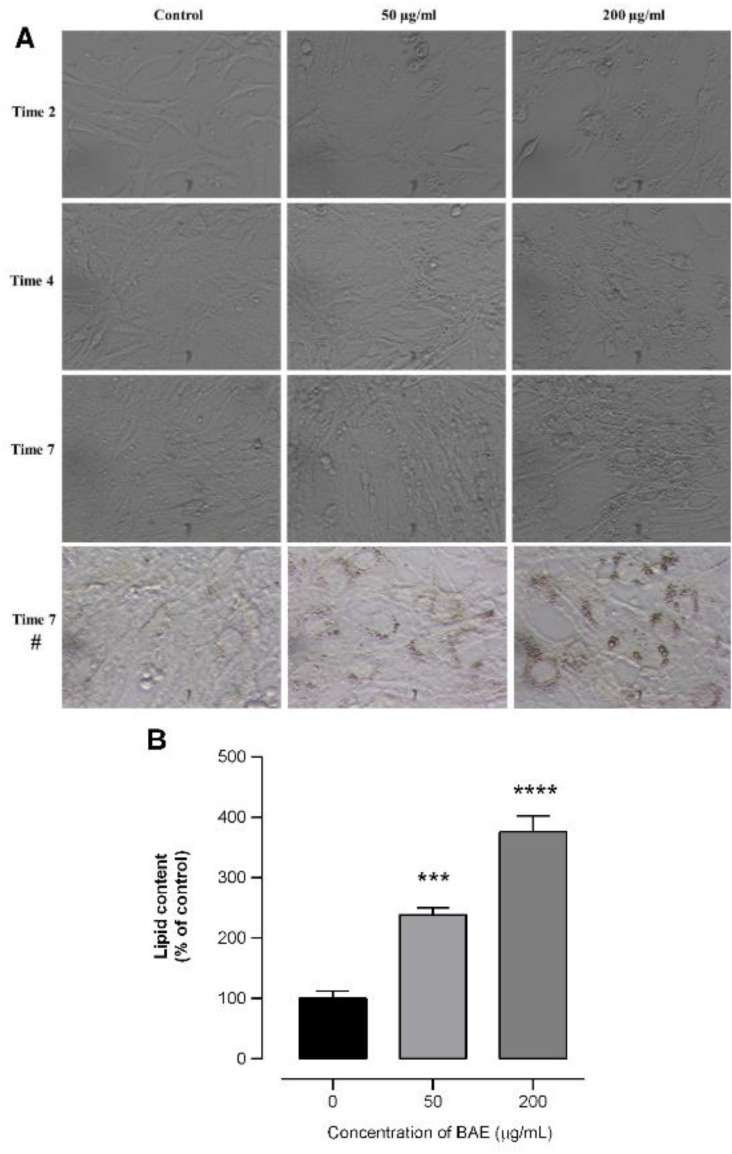
Effect of *Baccharis articulata *aqueous extract (BAE) on the fat-storing phenotype of murine hepatic stellate (GRX) cells. Panel A shows representative images of the accumulation of lipid droplets in GRX cytoplasm, which increased after treatment with BAE, as compared to the control. In panel B, the results of lipid quantification are shown as the absorbance values obtained for ORO, adjusted for the number of cells. Results are expressed as mean ± SEM (n=4) analyzed by ANOVA followed by Bonferroni's post-hoc test. *** *p* < 0.001; **** *p* < 0.0001: control vs. treated cells. 7 days #: GRX cells stained with ORO.

To evaluate the antifibrotic potential of BAE and chlorogenic acid, the MTT cell viability assay was used to study their biocompatibility on the GRX cell line. As shown in Fig.3(Fig. 3) (panel A), there was no significant cytotoxicity in GRX cell viability at any of the concentrations of BAE tested after 24 h of treatment, with viability maintained above 80 %. Therefore, intermediate concentrations of 10, 50, and 200 µg/mL were used in the following *in vitro* experiments to evaluate the antifibrotic effects of BAE. In addition, the extract reduced cell proliferation after seven days of treatment at 200 µg/mL (panel B), whereas no significant effects were observed for chlorogenic acid at 50 µg/mL (panel C). GRX cells cultured in a standard culture medium normally grow in monolayers and share an elongated appearance associated with the presence of a few lipid droplets (Hermann and Matter, 2007[31]). Morphological modifications were clearly visible from day two to seven of treatment, when BAE-treated GRX cells displayed a more polygonal shape, with the accumulation of lipids in the cytoplasm being noticeable at both concentrations tested, as shown in the qualitative images during cell treatment using an inverted light microscope (Fig. 4(Fig. 4), panel A). Lipid accumulation significantly increased in cells treated with the extract, whereas the myofibroblastic morphology of the control group was preserved. After the seventh day of treatment, lipid droplets were stained and quantified. GRX cells treated with 50 µg/mL presented 2.4 times more lipid droplets than control cells, while incubation with 200 µg/mL BAE led to 3.8 times increase (Fig. 4(Fig. 4), panel B).

The accumulation of lipid content in the cytoplasm indicates that HSC are in a quiescent state, have a fat-storing phenotype, and accumulate retinol (Bastos et al., 2023[6]). The results revealed that treatment with BAE had an antiproliferative effect and induced fat accumulation in the cytoplasm, demonstrating that the extract was able to deactivate GRX cells from the fibroblastic phenotype. It can be suggested that the *B. articulata* aqueous extract displayed an effect similar to that of crude extracts obtained from *Baccharis anomala* leaves (popularly known as “cambará-de-cipó”) after a three-day treatment using the same cell line (Basso et al., 2019[5]). For both treated groups, the protective effect was correlated with the presence of phenolic compounds, of which chlorogenic acid was one of the main components in the plant extracts.

### Effects of BAE and chlorogenic acid on the collagen deposition from GRX cells

Although the inhibition of cell proliferation and presence of lipid droplets are considered important factors related to the deactivation of stellate cells, one of the most well-studied components of hepatic fibrosis is the production of type I collagen (Chen et al., 2017[18]). Upon activation, HSCs become the main source of collagen in the liver, resulting in the secretion of ECM proteins and matrix metalloproteinases, leading to alterations in the architecture and physiological functions of the liver (Puche et al., 2013[45]; Zhang et al., 2016[63]). Therefore, modulation of stellate cell processes plays a key role in resolving hepatic fibrotic events.

**Figure 5:**
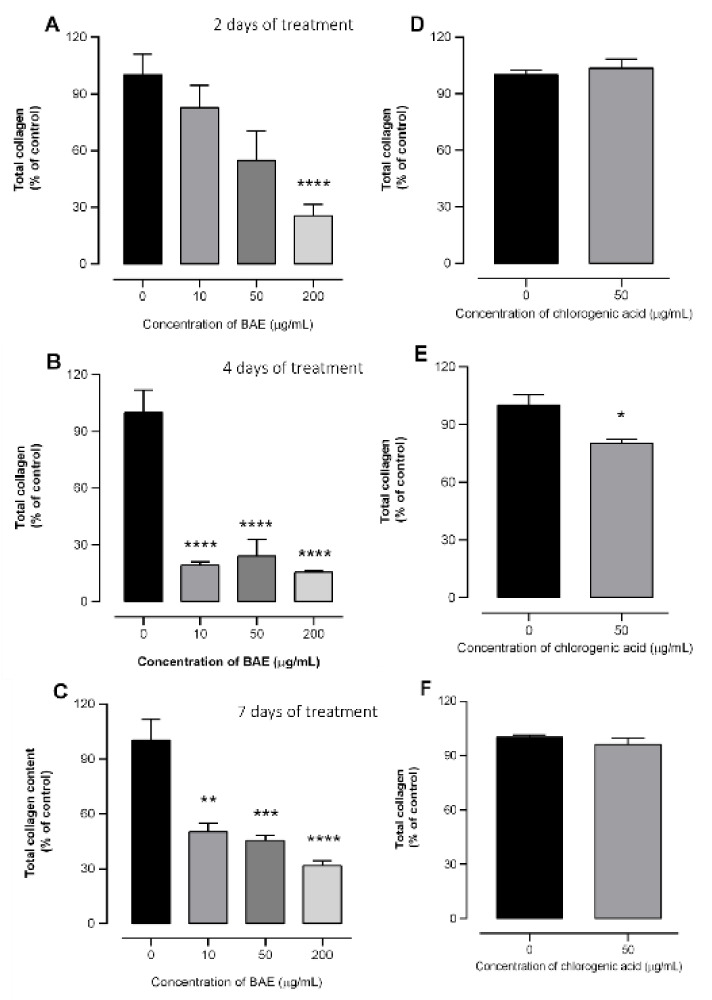
Effects on the total collagen content in murine hepatic stellate (GRX) cells supernatants after 2-, 4- and 7-day treatments with *Baccharis articulata *aqueous extract (BAE) (panels A, B, and C, respectively) and chlorogenic acid (panels D, E, and F, respectively). Results are shown as mean ± SEM (n = 4) analyzed by ANOVA followed by Bonferroni's post-hoc test. * *p* < 0.05, ** *p* < 0.01, *** *p* < 0.001 **** *p* < 0.0001: control *vs.* treated cells

The effects of BAE (10, 50, and 200 µg/mL) and chlorogenic acid (50 µg/mL) on the collagen content secreted by GRX cells were tested after 2, 4, and 7 days of treatment (Fig. 5(Fig. 5)). After 2 days of incubation, BAE at the highest concentration significantly reduced collagen content by approximately 74.5 % compared to the control (panel A). However, on the 4^th^ day all of the extract concentrations presented the maximum effect, showing reductions of 80.8 %, 75.8 %, and 84.3 % in collagen content at 10, 50, and 200 µg/mL, respectively (panel B). The effect of BAE on the inhibition of collagen deposition was sustained until the 7^th^ day of treatment, as observed in panel C, and was 49.7, 54.5, and 68.5 % lower in the culture medium of GRX cells treated with BAE (10, 50, and 200 µg/mL, respectively) than in the control, indicating an antifibrotic effect of the extract. Conversely, treatment with chlorogenic acid decreased cell collagen deposition only on the 4^th^ day of incubation at 50 µg/mL (19.6 %, panels D and E), but this effect was not sustained until the end of the experiment (panel F). In addition to deactivation of HSCs, previous studies have shown that many well-established mechanisms are related to the anti-liver fibrosis activity of chlorogenic acid. Among them, scavenging of excess free radicals, inhibition of apoptosis, and inflammation triggered by hepatotoxic agents through the modulation of TGF-β1 and VEGF secretion, as well as the toll-like receptor 4 and Nrf2/PPARα signaling pathways (Kim et al., 2018[35]; Nguyen et al., 2024[42]; Shi et al., 2009[50]; Shi et al., 2016[51]; Yang et al., 2017[60]). Moreover, the effect of chlorogenic acid on HSCs assessed in *in vivo* and *in vitro *studies associated its antifibrotic effects with a reduction in the expression of important proteins related to hepatic fibrosis, such as α-smooth muscle actin (α-SMA) and type I collagen, by complex networks of signaling pathways (Yang et al., 2017[60]) and reduction of oxidative stress (Shi et al., 2016[51]). It is widely known that complex mixtures of plant-derived bioactive compounds often present higher efficacy than purified molecules owing to the important interactions between compounds within the mixture (Caesar and Cech, 2019[15]). Therefore, the results presented in the present study suggest that this phenolic compound may be important for the antifibrotic effect of BAE; however, there are other bioactive compounds present in the extract that may contribute to the above-mentioned bioactivity.

### Effects of BAE on the modulation of the TGF-β1 and PAI-1 expressions in GRX cells 

**Figure 6:**
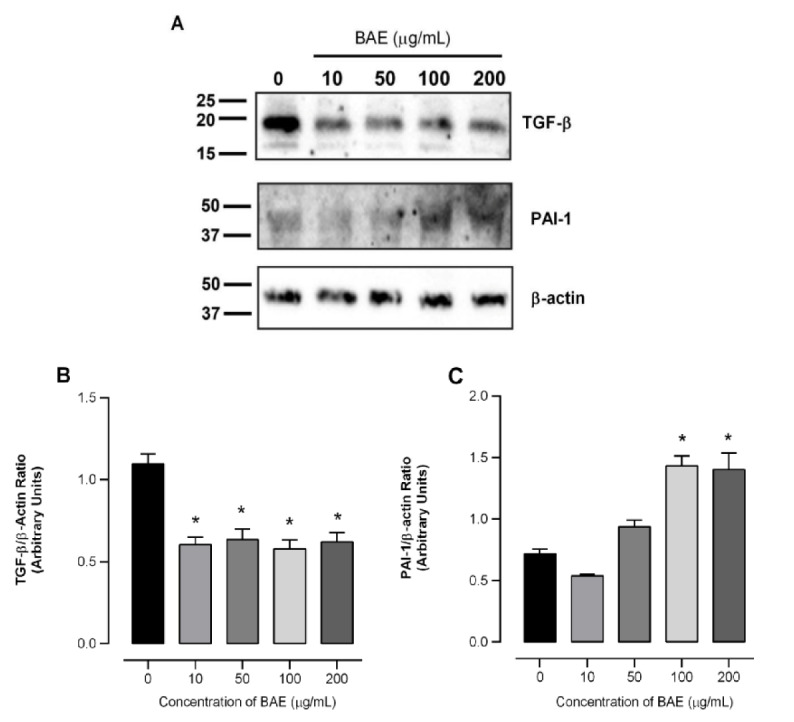
Effects of *Baccharis articulata *aqueous extract (BAE, 10 - 200 µg/mL) on the expression of fibrosis markers in murine hepatic stellate (GRX) cells via immunoblot detection after 24 h of treatment. Panel A shows band gels, while panels C and D represent band densities for the analysis of TGF-β and PAI-1 markers, respectively. Results are shown as mean ± SEM (n = 4) analyzed by ANOVA, followed by Bonferroni's post-hoc test. * *p* < 0.05: control *vs.* treated cells.

The ability of the aqueous extract of *B. articulata* to regulate both TGF-β1 and PAI-1 expression during fibrosis was investigated. Western blot analysis showed that pretreatment with BAE (10-200 µg/mL) significantly reduced TGF-β1 expression in GRX cells after seven days of treatment (Fig. 6(Fig. 6)). Under treatment, the observed reductions ranged from 53 to 58 % compared to the control (panels A and B), and were consistent with our previous findings on GRX cell deactivation and antifibrotic activity. Moreover, Fig. 6C(Fig. 6) shows that PAI-1 expression was almost two-fold higher in cells treated with the extract than in control cells. Given that TGF-β1 is considered a pivotal cytokine that directly correlates with the activation of HSCs and the subsequent accumulation of ECM proteins, it is plausible to conclude that it plays a significant role in the progression of chronic hepatic diseases (Dewidar et al., 2019[25]; Lee et al., 2020[37]). Thus, targeting its expression may be a promising strategy for inhibiting liver fibrosis (Bataller and Brenner, 2005[7]). 

The results revealed that the extract induced a fat-storing phenotype in GRX cells, and the antifibrotic effect was likely related to the suppression of active TGF-β1. These findings agree with previous studies indicating that inhibition of profibrogenic cytokines results in an important mechanism for inducing the lipocyte phenotype and decreasing the expression of ECM proteins, such as α-SMA and type I collagen, in GRX cells (Bastos et al., 2023[6]; Bitencourt et al., 2012[8]). The presence of chlorogenic acid in the aqueous extract of *B. articulata* may be suspected as possible contributor to the observed mechanism of action. The basis for such a premise is based on previous studies reporting this caffeoylquinic acid as a potent negative regulator of the TGF-β1 signaling pathway in hepatic stellate cells LX2 (Yang et al., 2017[60]) and in fibrotic rats following CCl_4_-hepatic injury (Shi et al., 2009[50]). 

**Figure 7:**
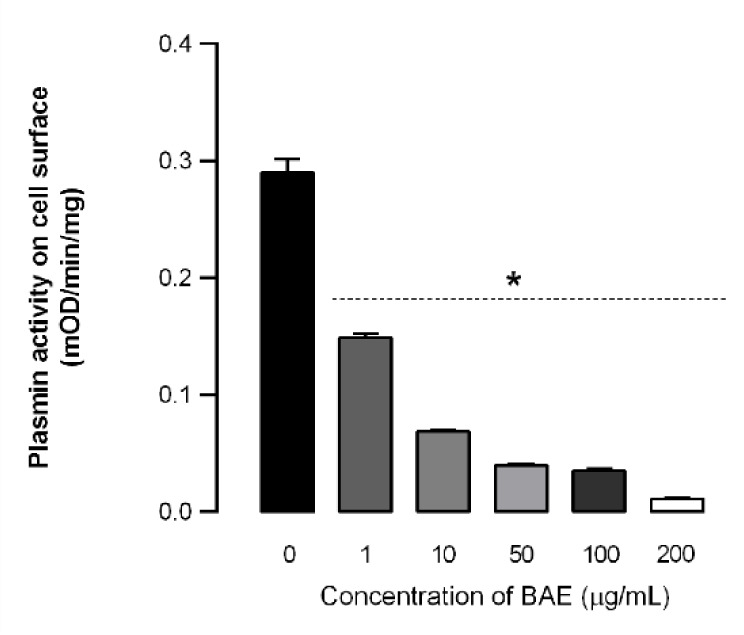
Effects of *Baccharis articulata *aqueous extract (BAE) on plasmin generation in murine hepatic stellate (GRX) cell monolayers cultured in the presence of plasminogen (0.1 mg/mL) and treated with the extract (1 - 200 µg/mL) for 24 h. Results are shown as mean ± SEM (n = 4) analyzed by ANOVA followed by Bonferroni's post-hoc test. * *p* < 0.05: control *vs.* treated cells.

As the extract affected plasmin generation by upregulating PAI-1 expression, the next step was to evaluate the conversion of plasminogen to plasmin on the surface of treated cells. As shown in Fig. 7(Fig. 7), BAE inhibited plasmin formation in a dose-dependent manner, with a decrease in plasmin activity on the cell surface ranging from 48.6 to 96.2 %. It has been previously established that HSC-T6 and LX-2 stellate cell lines may regulate the synthesis and degradation of ECM components and subsequent matrix remodeling by modulating the expression of PAI-1 and the plasminogen-activating system (Hermann and Matter, 2007[31]; Leyland et al., 1996[39]), although there are conflicting results regarding their potential risks or benefits. 

PAI-1 is the main inhibitor of both tissue-type (tPA) and urokinase-type (uPA) plasminogen activators and is a key regulator of fibrinolysis by plasmin (Sultana et al., 2019[55]). While there is evidence that PAI-1 expression is elevated and that its excessive expression inhibits ECM degradation, it also plays an important role in fibrogenic disorders (Hu et al., 2023[32]). However, findings regarding the role of PAI-1 in liver fibrosis are still conflicting. The expression of PAI-1 has demonstrated a protective effect against CCl_4_-induced hepatic fibrosis in mice (von Montfort et al., 2010[59]). In this study, high concentrations of BAE increased PAI-1 expression after seven days of incubation. To the best of our knowledge, this is the first study to investigate the expression of PAI-1 in GRX cells.

**Figure 8:**
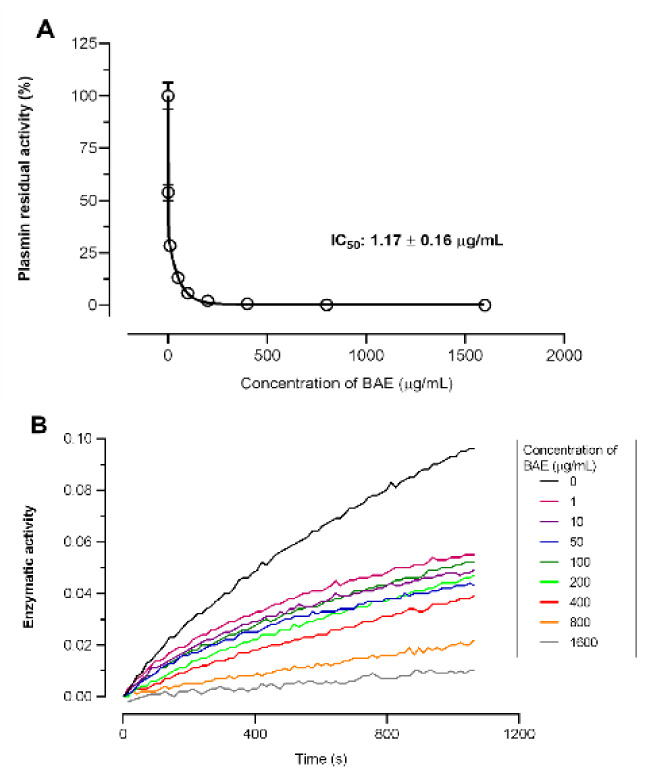
Effect of *Baccharis articulata *aqueous extract (BAE, 0 - 1600 µg/mL) on plasmin inhibition. Panel A shows the progress curves of the kinetics of plasmin inhibition by the extract. Panel B shows the residual activity plotted against the BAE concentration for IC_50_ calculations.

Lastly, the evaluation of plasmin enzyme kinetics showed that BAE incubation can directly inhibit plasmin activity also in a dose-dependent manner, with an IC_50_ of 1.17 ± 0.16 μg/mL (Fig. 8(Fig. 8), panels A and B). As the phenotypic changes in GRX cells and decreased collagen accumulation in the ECM were more pronounced at the same concentration, the inhibition of plasmin formation appears to be beneficial. Based on these results, the effects of BAE on the plasminogen-activating system were evaluated, which resulted in decreased plasmin formation on the surface of GRX cells. Additionally, an enzymatic assay demonstrated that BAE directly inhibited plasmin activity. After liver injury, plasmin-mediated activation causes degradation of the normal ECM and activation of HSCs by activating TGF-β1, which is secreted in latent form (Leyland et al., 1996[39]). It is possible that the inhibition of the conversion of plasminogen to plasmin or plasmin activity may prevent the formation of active TGF-β1 *in vitro. *Since previous studies evaluating the effect of polyphenols on plasmin and plasminogen activation systems did not detect any inhibitory activity of chlorogenic acid, BAE activity appears to be related to the different molecules present in the preparation (Ogston et al., 1985[43]). Further investigations are required to evaluate the mechanisms of action of BAE and the bioactive compounds responsible for its effects on the GRX cells.

## Conclusions

In this study, the aqueous extract prepared from the wing stems of *B. articulata*, a medicinal plant species widely used in Southern Brazilian folk medicine for digestive and hepatic disorders, was studied for its liver antifibrotic properties *in vitro*. HPLC-DAD-MS/MS of BAE revealed an abundance of phenolic compounds, including flavonoids, diterpenes, and several caffeoylquinic acids such as chlorogenic acid. BAE induced phenotypic conversion of GRX cells and exerted an antifibrotic effect by decreasing the collagen content in the ECM as well as the expression of TGF-β1. As it was the first time that the plasminogen activation system was studied using GRX cells, more studies are necessary to elucidate the role of PAI-1; however, it seems that inhibition of plasmin activity may play an antifibrotic role in this *in vitro *model. Finally, this study suggests that *B. articulata* has potential therapeutic activity against liver fibrosis, partially owing to the presence of caffeoylquinic acids. Further investigations are required to elucidate the mechanisms of action and the underlying active components. 

## Declaration

### Authors' contributions 

Eduardo Luis Konrath and Markus Berger conceived and designed the study. Daiana Daniele Boeff, Markus Berger, Mariana Koetz, Pamela Zanon, Alicia da Costa Pereira and Katyuce Souza Farias conducted the research and assisted with methodology development. Daiana Daniele Boeff wrote the first draft of this manuscript. Daiana Daniele Boeff, Markus Berger, Paula Barros Terraciano, Carlos Alexandre Carollo and Eduardo Luis Konrath interpreted the data. All the authors were involved in revising the manuscript and provided their approval for the final version submitted. The corresponding author certifies that all listed authors meet the required authorship criteria and that no qualified individuals have been left out.

### Acknowledgements

Authors are thankful to the Centro de Pesquisa Experimental (CPE-HCPA UFRGS) for providing help and technical support. This study was financed in part by the Coordenação de Aperfeiçoamento de Pessoal de Nível Superior - Brasil (CAPES) - Finance Code 001, and the Fundação de Amparo à Pesquisa no Estado do RS (FAPERGS, Project 24/2551-0001387-3).

### Conflict of interest

We confirm that there are no known conflicts of interest associated with this publication and there has been no significant financial support for this work that could have influenced its outcome.

### Data sharing 

The data generated during the current study are available from the corresponding author (Eduardo Luis Konrath) upon reasonable request.

### Using Artificial Intelligence (AI) 

Authors used artificial intelligence (AI)-assisted technologies to check grammar errors.

## Figures and Tables

**Table 1:**
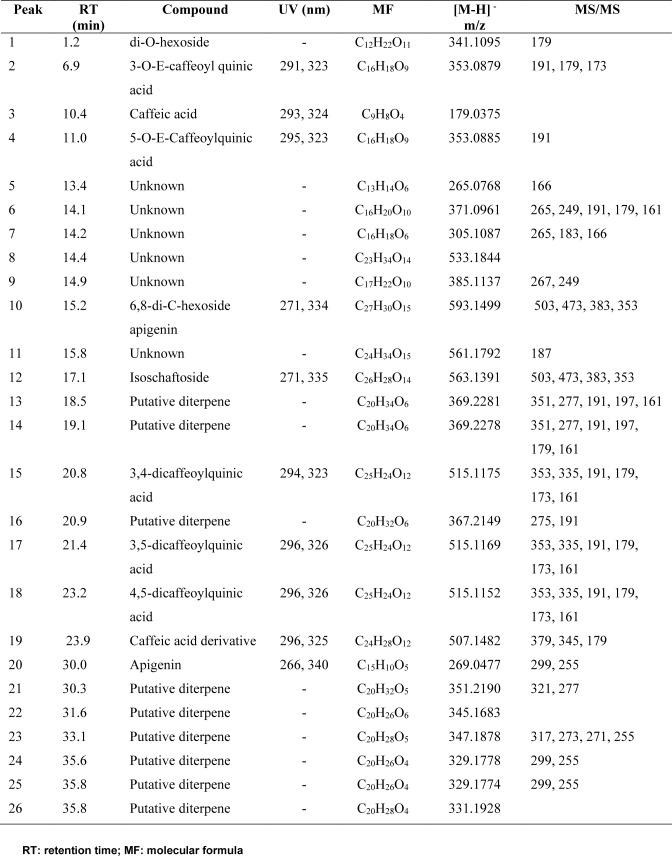
Compounds annotated from Baccharis articulata aqueous extract (BAE) by LC-DAD-MS/MS

**Figure 1:**
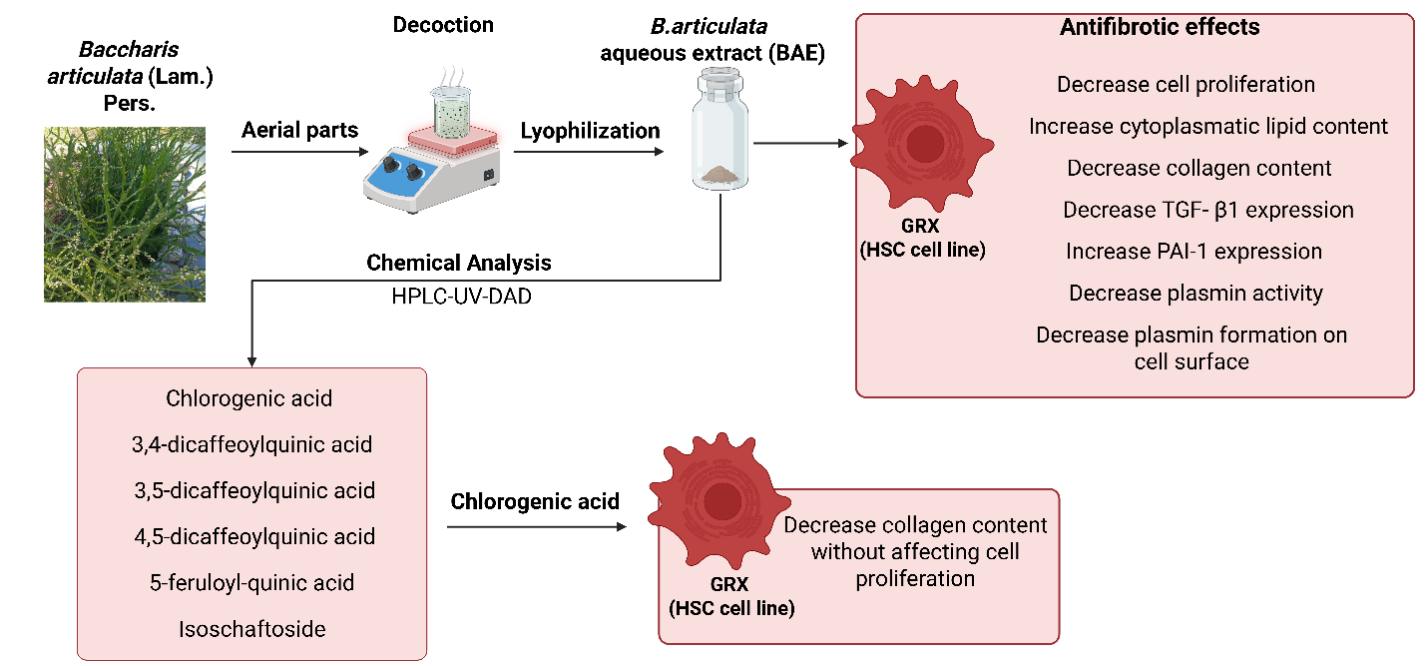
Graphical abstract
